# Acute ingestion of caffeinated chewing gum reduces fatigue index and improves 400-meter performance in trained sprinters: a double-blind crossover trial

**DOI:** 10.1080/15502783.2024.2414871

**Published:** 2024-10-10

**Authors:** Yi-Jie Shiu, Che-Hsiu Chen, Wu-Shiun Tao, Hui-Fang Nai, Chen-Yi Yu, Chih-Hui Chiu

**Affiliations:** a National Taiwan Normal University, Physical Education and Sport Sciences, Taipei, Taiwan; b National Taiwan University of Sport, Department of Sport Performance, Taichung, Taiwan; c National Taiwan University of Sport, Department of Ball Sport, Taichung, Taiwan; d National Taiwan University of Sport, Graduate Program in Department of Exercise Health Science, Taiwan

**Keywords:** α-amylase, ergogenic aids, athletes, nutrition

## Abstract

**Background:**

This study investigated the effects of caffeinated chewing gum on fatigue index and 400-meter performance in trained sprinters.

**Methods:**

Nineteen participants (age: 20.9 ± 1.0 years; height: 175.6 ± 4.9 cm; mass: 66.5 ± 5.6 kg; training age: 7.9 ± 1.0 years) were randomly assigned to either a caffeine trial (CAF) or a placebo trial (PL) using a double-blind, randomized crossover design. The participants in the CAF trial chewed a gum containing 3 mg/kg of caffeine for a period of 10 minutes, while those in the PL trial chewed a gum containing a placebo with no caffeine. Following a 15-minute period of rest, the fatigue index was tested by six maximal 35-meter sprints with a 10-second rest between efforts. After this, at least 30 minutes of rest was permitted, during which time the participants engaged in brief warm-up activities prior to the commencement of the 400-meter sprint test. Saliva samples were collected before chewing gum, before the fatigue test and before 400-meters sprinting.

**Results:**

The fatigue index was significantly lower in the CAF trial compared to the PL trial (CAF: 8.1 ± 2.5%; PL: 9.6 ± 4.8%; *p* = 0.046, Cohen’s d = 039). The CAF trial demonstrated significantly lower sprint time for the 300–400 meter segment (CAF: 14.73 ± 1.35 seconds; PL: 15.23 ± 1.30 seconds; *p* = 0.019, Cohen’s d = 0.37) and total sprint time compared to the PL trial (CAF: 53.87 ± 2.88 seconds; PL: 54.68 ± 3.37 seconds; *p* = 0.003, Cohen’s d = 0.27). Saliva caffeine and α-amylase concentration were significantly higher in the CAF trial compared to the PL trial (*p* < 0.05).

**Conclusion:**

The present study demonstrated that caffeine gum supplementation prior to exercise significantly reduced the fatigue index and increased the capacity to maintain speed, particularly in the final 300 to 400 meters, as well as enhancing 400-meter sprint performance.

## 1. Introduction

Short-distance races are the most popular events at the Olympic Games and the World Athletics Championships. Successful short-distance sprinting requires the ability to accelerate, maintain maximum speed, and decelerate. These factors influence the performance in short-distance race [1]. For the 400-meter event, the race demands sustained maximum velocity throughout the event [2]. Approximately 59% of the energy utilized by male 400-meter runners originates from the anaerobic energy system [3]. Therefore, anaerobic capacity is an important criterion for differentiating elite players from the sub-elite players [4]. Enhancing anaerobic capabilities is therefore pivotal for optimizing 400-meter sprint performance.

Caffeine (1,3,7-trimethylxanthine) is a widely consumed nutritional supplement, particularly among athletes. Caffeine supplementation has been demonstrated to enhance athletic performance. Physiologically, the ingestion of caffeine prior to exercise presents numerous mechanisms for enhancing anaerobic performance. These mechanisms are comprised of antagonized adenosine receptors, reducing exercise fatigue, increases sarcoplasmic reticulum calcium ions release, maintains sodium-potassium ATPase (Na+/K±ATPase) activity, and stimulates glycolysis [5,6]. These mechanisms suggest caffeine’s potential to improve anaerobic capacity. Therefore, the International Society of Sports Nutrition recommends caffeine supplementation of 3–6 milligrams per kilogram of body weight, administered one hour before exercise, to enhance anaerobic power performance [7].

Another factor that may affect the ability to sprint over short distances is the activity of the sympathetic nervous system. A recent study has indicated that the sympathetic excitability of the brain plays a pivotal role in attaining optimal sprinting capabilities [8]. Enhanced sympathetic activation during exercise has been found to be effective in reducing fatigue during exercise and increasing energy output at maximum exercise intensity [9]. Furthermore, evidence indicates that sympathetic activation impacts arterial blood pressure and blood flow regulation [8]. It can be surmised from these mechanisms that an increase in sympathetic nerve activity prior to exercise may be an important factor in improving anaerobic capacity and enhancing performance in the 400-meter race. To understand the state of sympathetic nerve activation, one can measure the electrode signal from the heart, α-Amylase in saliva, or the concentration of catecholamines in plasma [8]. Studies have shown that caffeine supplementation before exercise is effective in increasing sympathetic nerve activity [10]. However, it remains to be investigated whether caffeine supplementation is effective in enhancing sprinting capacity in the 400-meter sprint may be in part attributable to this mechanism.

The time required for caffeine supplements, including coffee, energy drinks, and capsules, to reach their maximum caffeine levels in the bloodstream is typically approximately an hour. For those seeking a rapid caffeine uptake, caffeine gum may be a viable alternative. The rate of absorption of caffeine from caffeinated chewing gum is faster than that of caffeine capsules, due to the fact that the former is absorbed via the buccal mucosa [11]. A previous study suggested that the ingestion of caffeinated chewing gum containing 3 mg/kg body weight for a period of 10 minutes, followed by a 15-minute rest period, resulted in the complete absorption of caffeine from the gum and the highest concentration of caffeine in the blood plasma [12]. A recent systematic review and meta-analysis study by Barreto et al.. (2023) found that chewing caffeine gum containing ≥3 mg/kg body mass 15 minutes before exercise effectively enhances endurance, muscular strength, and power performance [13]. Other studies have demonstrated the effectiveness of 3 mg/kg body mass caffeine gum in improving lower limb strength [14] and reducing fatigue index during repetitive sprints [15]. However, the relationship between caffeinated chewing gum and CMJ heights remains inconsistent in current study. Given the potential correlation between CMJ height and sprint acceleration [16], further investigation in this area is warranted. In addition, no studies have been conducted to examine the relationship between sympathetic nerve activity and 400-meter sprint performance. As a result, the aim of this study was to examine the impact of chewing caffeine-containing gum on sympathetic nerve activity, fatigue index, and 400-m sprint performance.

## 2. Methods

### 2.1. Study design

**Figure 1.**
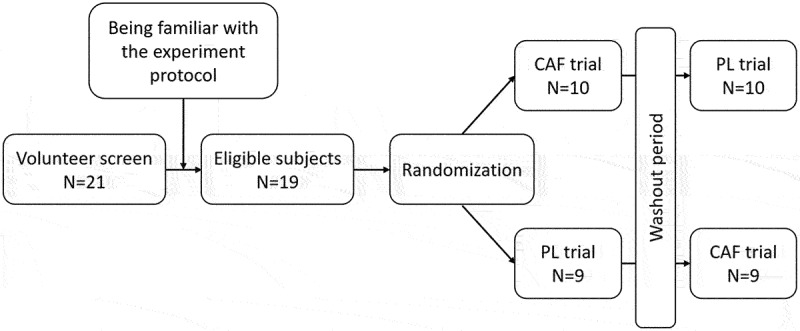
CONSORT diagram and study design.

This study investigated the effect of caffeine gum on the 400-meter sprinting performance in elite sprinters using a randomized, cross-over, double-blind design. Participants were randomly assigned to either chew a caffeinated gum (CAF trial) at a dosage of 3 mg/kg body weight or a placebo gum (PL trial). Following a seven-day washout period, they switched to the other gum for the crossover trial (Figure 1). Prior to the commencement of the formal experiment, all participants underwent one or two familiarization sessions with the anaerobic sprints test in order to ensure that they understood the procedures and could perform them correctly. The primary outcome measure was 400-meter sprint performance, while the secondary outcome was fatigue index. The study commenced on 8 April 2024 and concluded on 1 July 2024.

### 2.2. Participants

Twenty-one professionally trained track and field male sprinters were recruited for this study. After two participants withdrew due to personal reasons, the final results of 19 participants (age: 20.9 ± 1.0 years; height: 175.6 ± 4.9 cm; mass: 66.5 ± 5.6 kg; training age: 7.9 ± 1.0 years) were analyzed. The inclusion criteria for this study were: (a) more than 6 years of professional sprint training; (b) more than 6 months of continuous training; (c) more than 3 months of recovery from sports injuries such as strains and sprains. The exclusion criteria were: (a) non-professionally trained track and field sprinters; (b) lack of regular training in the past 6 months; (c) less than 3 months of recovery from a sports injury, or present epilepsy, hypertension, hyperlipidemia, heart disease, arthritis, osteoporosis, or brain injury; (d) a history of caffeine allergy. This study received approval from the Institutional Review Board of Jen-Ai Hospital – Dali Branch (202300071B0) and registered in ClinicalTrials.gov (Date: 05/24/2024; ID “NCT06430996;” https://register.clinicaltrials.gov). This study was conducted following the Declaration of Helsinki.

### 2.3. Sample size calculation

We used G*Power software (version 3.1.9.4, Universität Düsseldorf, Germany) [17] to determine the required sample size. The calculation was based on an alpha level of 0.05 and a correlation coefficient of 0.80. Based on data from a previous investigation [15], a large effect size (Cohen’s d = 1.00) in the fatigue index after ingestion of caffeinated chewing gum. The analysis indicated that a sample size of 9 would be sufficient for the detection of a difference between trials. In a previous study, a sample size of 13 was sufficient to detect statistically significant differences in 100-m sprint test results following acute caffeine supplementation in male collegiate sprinters [18]. The recruitment of 19 participants in this study should be sufficient to elucidate the statistical discrepancies.

### 2.4. Protocol

Prior to the first main test, the dietitian instructed participants on permissible food choices, emphasizing the avoidance of sugar, caffeine, and sugary beverages. Participants were required to record their diet and training protocol for the first three days and maintain the same diet and training protocol until the next trial. On the day of the trial, participants were provided with the same breakfast and lunch to ensure they consumed the same amount of energy before the test. Breakfast was served at 08:00 am and lunch at 12:00 pm. The meals were designed to provide a consistent macronutrient profile, with an average of 12.8 ± 1.5% protein, 40.7 ± 15.3% carbohydrate, and 28.6 ± 5.2% lipid, and an average energy intake of 1232.7 ± 132.2 kcal.

All experiments started at 03:00 pm. Participants reported to the track and field stadium and rested quietly for 15 minutes. Participants were first asked about their rating of perceived exertion (RPE) scores. The RPE score was recorded using a 1–10 scale [19]. Saliva samples were collected for subsequent biochemical analyses. The fingertip lactate concentration was collected and analyzed immediately using lactate chemistry analyzer (KDK Corporation, Siga, Japan). Participants then consumed either caffeinated gum (CAF) or placebo gum (PL) for 10 minutes. After spitting out the chewing gum, a 15-minute dynamic warm-up was conducted. This experimental protocol has been demonstrated to optimize blood caffeine levels [12], enhance lower limb muscle strength [14], and reduce fatigue [15]. Saliva samples were collected again, followed by a countermovement jump (CMJ) test. It has been demonstrated that there is a correlation between the height of the countermovement jump (CMJ) and the speed of acceleration [20]. After a 10-minute rest, participants complete an anaerobic sprint test. A final saliva samples were collected post-exercise, followed by a 30-minute recovery period and a brief warm-up before the 400-meter sprinting test. The fingertip lactate concentration and RPE score were record on baseline, before and after 400-meter sprinting test.

### 2.5. Outcome measure

The countermovement jump test was evaluated utilizing a jumping mat (Fusion Sport, Coopers Plains, QLD, Australia). Participants maintained a static standing position on the jumping mat with arms folded at the waist. After receiving instructions from the instructor, participants maintained a position with arms akimbo and performed a rapid squat to about 90 degrees of knee flexion before executing a maximal vertical jump. Hip and knee extension were maintained throughout the jump. Trials with knee flexion less than 90 degrees or foot contact were excluded. Participants completed three trials with 1-minute rest between each trial, and the average value was calculated. Similar experimental procedures have been used in previous study [15].

The anaerobic sprints test was employed the running anaerobic sprint test (RAST) [21], which involved six maximal 35-meter sprints with a 10-second rest between efforts. Peak, average, and minimum power, as well as fatigue index, were determined [21]. Light gates (Microgate®, Whitty, Italy) were positioned at the start (0 m) and finish (35 m) of the track, with a sufficient margin for a 10-second buffer zone. Subsequently, participants completed six maximal 35-meter sprints with 10-second interval, followed by a 30-minute recovery period before subsequent testing. The power of effort for each sprints was calculated using the participant’s body weight and sprint time. The calculation formula was: power = (body mass * distance^2^)/time^3^. The maximum value was defined as maximum power, while the minimum value was defined as minimum power. The calculation formula for fatigue index (%) was: (maximum power – minimum power/total time) * 100.

The 400-meter sprint test was conducted on the track and field stadium. Light gates were placed at the start, 100-meter, 200-meter, and 300-meter marks, and the finish line. Once the participant is prepared, a three-point start was initiated, and participant were requested to complete the test at maximal effort. The light gates used in this study have an accuracy of 0.001 seconds.

### 2.6. Saliva sample collection and analysis

All participants were seated and instructed to thoroughly rinse their mouths with 30 ml of sterile distilled water prior to sample collection. Following a 10-minute resting period, saliva samples were collected into sterile plastic containers and immediately stored at − 80 °C. Saliva caffeine and α-amylase concentration were analyzed using enzyme-linked immunosorbent assay (ELISA) with commercial reagents (Neogen Corporation, Kentucky, USA; Salimetrics LLC, State College, PA, USA). All samples were assayed in triplicate, with intra-assay coefficients of variation (CVs) for caffeine and α-amylase of 4.3% and 5.2%, respectively.

### 2.7. Caffeine and placebo gum

The caffeinated chewing gum used in this study (Military Energy Gum, Arctic Mint flavor; Stay Alert, Chicago, USA) has been used in previous studies [12,15]. Each piece of gum contains approximately 5 grams and 100 milligrams of caffeine. The placebo gum was a commercially available blue mint gum (Lotte Gum Mint., Ltd., Tokyo, Japan). To achieve a 3 mg/kg body weight caffeine dose, all chewing gums were crushed, ground, blended, reshaped, and flavored with 0.3 g of peppermint flavoring powder. This process ensured that they were similar to the original chewing gums in terms of appearance, color, flavor, weight and size. This method has been validated through a double-blind study [15].

### 2.8. Statistical analysis

All data are presented as means ± standard deviations. The Shapiro – Wilk test was used to assess the normality of the data. The jump height of CMJ, anaerobic sprints test, and 400-meter sprinting test between the two trials were analyzed using paired t-tests. The saliva caffeine, α-amylase, fingertip lactate blood concentration and RPE were analyzed using two-way ANOVA with repeated measures. If the interaction effect (trial  × time) were significant, the Bonferroni method was used to perform post hoc comparisons. Effect sizes were calculated using Cohen’s d and eta squared to quantify the magnitude of the observed effects. The power analysis for each data set was conducted using G*Power 3.1.9.6 software [17]. The significance level was set at α < 0.05.

## 3. Results

### 3.1. Baseline parameters

There were no significant differences in baseline RPE scores, blood lactate concentrations and α-amylase concentrations between the two trials (Table 1).

**Table 1.** Baseline parameters.

	CAF	PL	P
RPE	2.4 ± 0.8	2.5 ± 0.9	0.651
Saliva caffeine	0.0 ± 0.0	0.0 ± 0.0	1
Saliva α-amylase	133.8 ± 60.7	136.1 ± 61.4	0.820

Values are mean SD, *n* = 19. CAF, caffeine trial; PL, placebo trial; RPE, rating of perceived exertion.

### 3.2. Jump height of CMJ

**Figure 2.**
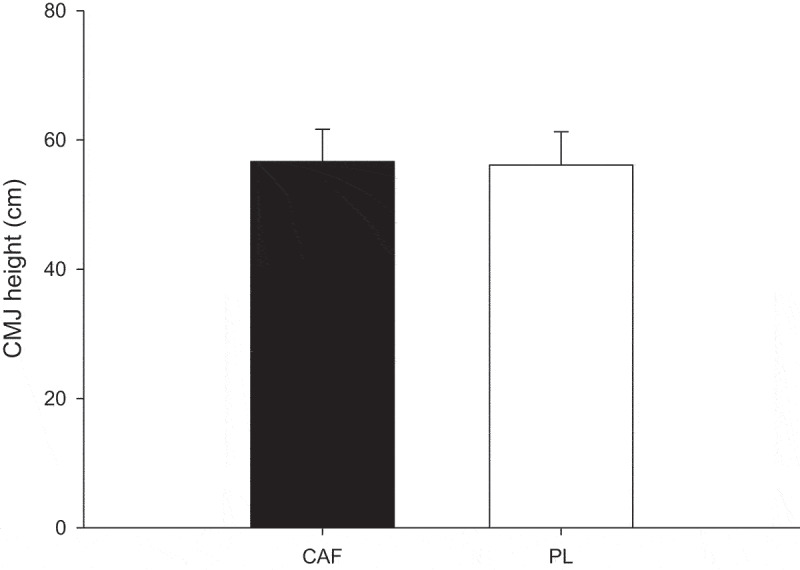
Jump height of CMJ. Values are mean SD, *n* = 19.

There was no significantly different on jump height of CMJ (*p* = 0.451) in the CAF trial compared to the PL trial (Figure 2).

### 3.3. Anaerobic sprint test

**Figure 3.**
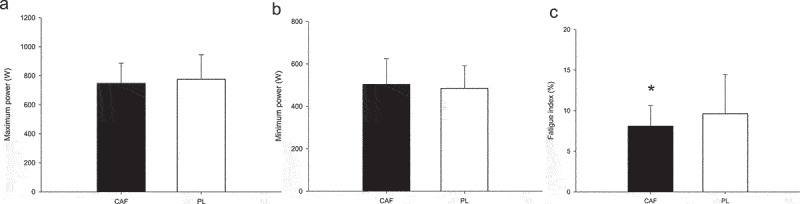
Anaerobic sprint test. Values are mean SD, *n* = 19. The maximum power (a), minimum power (b) and the fatigue index (c). * CAF was significantly lower than those for the PL.

Caffeinated chewing gum significantly reduced the fatigue index, as measured by the anaerobic sprint test, compared to the PL trials. No significant differences in maximum power (*p* = 0.133; Figure 3a) or minimum power (*p* = 0.161; Figure 3b) were observed between the CAF and PL trials. The fatigue index (Figure 3c) was significantly lower in the CAF trial compared to the PL trial (CAF: 8.1 ± 2.5%; PL: 9.6 ± 4.8%; *p* = 0.046, Cohen’s d = 039).

### 3.4. Saliva caffeine and α-amylase concentration

**Figure 4.**
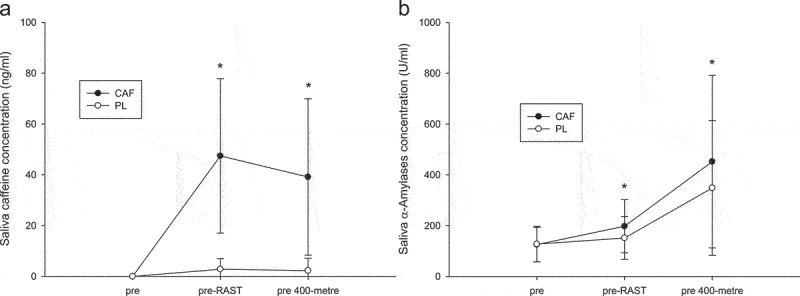
Saliva caffeine and α-amylase concentration. Values are mean SD, *n* = 19. The saliva caffeine concentration (a) and Salivaα-amylase concentration (b) * CAF was significantly higher than those for the PL.

There was an interaction effect (*p* < 0.001; η2 = 0.638; Figure 4a), an effect of trial (*p* < 0.001; η2 = 0.658) and time (*p* < 0.001; η2 = 0.703) for saliva caffeine concentration (ng/mL). Post-hoc analysis revealed that saliva caffeine concentrations were higher in the CAF trial than in the PL trial at pre-RAST (*p* < 0.001; Cohen’s d = 2.06) and pre 400-meter sprint (*p* = 0.001; Cohen’s d = 1.73). Similarly, there was an interaction effect (*p* = 0.031; η2 = 0.205; Figure 4b), an effect of trial (*p* = 0.021; η2 = 0.307) and time (*p* < 0.001; η2 = 0.547) for saliva α-amylase concentration (U/ml). Post-hoc analysis revealed that saliva caffeine concentrations were higher in the CAF trial than in the PL trial at pre-RAST (*p* = 0.001; Cohen’s d = 0.49) and pre 400-meter sprint (*p* = 0.049; Cohen’s d = 0.34).

### 3.5. The 400-metres sprint test

There was an interaction main effect (*p* = 0.035; η2 = 0.211), an effect of trial (*p* = 0.032; η2 = 0.328) and time (*p* < 0.001; η2 = 0.843) for the sprinting time in different segments. No significant differences in sprint time were observed between the CAF and PL trials for the 0–100 meter, 100–200 meter, and 200–300 meter segments. However, the CAF trial demonstrated significantly lower sprint time for the 300–400 meter segment (CAF: 14.73 ± 1.35 seconds; PL: 15.23 ± 1.30 seconds; *p* = 0.019, Cohen’s d = 0.37) and total sprint time (CAF: 53.87 ± 2.88 seconds; PL: 54.68 ± 3.37 seconds; *p* = 0.003, Cohen’s d = 0.27) compared to the PL trial (Table 2).

**Table 2.** The 400-meters sprint test.

	CAF	PL	*P* value	Cohen’s *d*
0-100 m	12.48 ± 0.43	12.57 ± 0.51	0.410	0.19
100-200 m	12.57 ± 0.46	12.69 ± 0.83	0.477	0.18
200-300 m	13.89 ± 0.90	14.04 ± 1.06	0.203	0.11
300-400 m	14.73 ± 1.35*	15.23 ± 1.30	0.019	0.37
Total	53.87 ± 2.88*	54.68 ± 3.37	0.003	0.27

Values are mean SD, *n* = 19. CAF, caffeine trial; PL, placebo trial. * CAF was significantly lower than those for the PL.

### 3.6. The lactate concentration and RPE score before and after 400-meter sprinting

There were no significant differences in RPE (trial × time, *p* = 0.451; trial, *p* = 0.508; time, *p* < 0.001) and blood lactate concentrations (trial × time, *p* = 0.691; trial, *p* = 0.607; time, *p* < 0.001) before and after the 400 m sprint between the two trials (Table 3).

**Table 3.** The lactate concentration and RPE score before and after 400-meter sprinting.

	CAF	PL
	lactate concentration (mmol/L)	
Before 400-meter sprinting	2.1 ± 0.9	2.1 ± 0.8
After 400-meter sprinting	14.8 ± 3.7	15.5 ± 4.2
	RPE score	
Before 400-meter sprinting	6.7 ± 1.8	7.0 ± 2.0
After 400-meter sprinting	9.2 ± 1.1	9.2 ± 1.2

Values are mean SD, *n* = 19. CAF, caffeine trial; PL, placebo trial; RPE, rating of perceived exertion.

## 4. Discussion

The present study demonstrated that caffeine gum supplementation (3 mg/kg) prior to exercise rapidly increased blood caffeine concentration, leading to a significant reduction in fatigue index and improved capacity to maintain 300–400 meter sprint velocity within a 400-meter race, as well as enhanced 400-meter sprint performance. Furthermore, the ingestion of caffeine in the form of a gum supplement has been demonstrated to effectively enhance the saliva α-amylase concentrations. The salivary α-amylase may be associated with activation of sympathetic nerves, which may be the underlying mechanism responsible for the observed reduction in the fatigue index.

The present study indicated that the enhanced speed maintenance observed in the CAF trial following caffeinated chewing gum consumption may be the primary contributor to improve 400-meter sprint performance. The reduction in fatigue attributed to caffeine supplementation likely stems from multiple mechanisms, including adenosine receptors antagonism, enhanced calcium ions release, maintained sodium-potassium ATPase (Na+/K±ATPase) activity, and increased sympathetic nerve activity [5,6]. Salivary α-amylase, employed as an indicator of sympathetic nerve activity, was significantly elevated in the CAF trial compared to the PL trial, supporting this hypothesis. A previous study has confirmed a positive correlation between elevated α-amylase levels and increased sympathetic nerve activity [22]. These findings suggest that chewing caffeinated gum for 10 minutes and resting for 15 minutes activated sympathetic nerve activity for a relatively short period of time. From this perspective, it appears that the activation of sympathetic nerves serves to reduce the fatigue index during exercise, which is a primary factor contributing to the enhanced performance of elite sprinters in the 400-meter sprint.

The function of sympathetic nerves and fatigue indices during exercise remains unclear in previous literatures. To the best of our knowledge, only one study has employed transcranial direct current stimulation (tDCS) to activate sympathetic nerves during exercise [9], resulting in reduced fatigue and enhanced energy output at maximal exercise intensity [9]. Other study has demonstrated that the activation of the sympathetic nervous system has been linked to the regulation of cardiovascular control and can be used to assess the fatigue index during voluntary isometric contraction [23]. Taken together, these findings, along with the α-amylase data from this study, suggest that the use of caffeinated chewing gum before exercise effectively increases sympathetic nerve activity. Also, this should also be framed as one possible contributing mechanism and not the only one that explains the improved performance. The present study provides innovative evidence that caffeine supplementation in the form of caffeinated chewing gum prior to exercise can rapidly activate sympathetic nervous effects, thereby decreasing the fatigue index and increasing the ability to maintain speed, especially in the last phase of 400-meter sprinting.

Previous research has indicated that caffeinated gum consumption can reduce fatigue [15], a finding corroborated by the present study. Liu et al. (2024) employed 15 trained basketball players as participants and discovered that the utilization of caffeinated gum chewing was an effective method for reducing the players’ fatigue index [15]. While this study extends these finding to 400-meter sprinting, it is the first to investigate the relationship between caffeine gum, sympathetic nerve activation, and sprint performance. Furthermore, the results corroborate the findings of the previous systematic review and meta-analysis of caffeinated chewing gum [13], while also suggesting an alternative potential mechanism through which sympathetic nerve activity may influence fatigue index and the 400 m sprint. No significant difference was observed between the two trials with regard to the RPE and blood lactate data. This indicates that all participants were in a comparable physiological state prior to the 400 m sprint and exerted their maximum effort during the sprint. The observed enhancement in 400-meter sprint performance, particularly in the final sprint segment, suggests that caffeine gum’s ability to reduce fatigue and potentially increase sympathetic nerves activity may be key mechanisms underlying these improvements.

The present study revealed no significant enhancement in CMJ performance between the CAF and PL trial. In addition, no discernible differences in sprinting ability were observed during the initial 100 meters of sprinting. A previous review study indicated that caffeine supplementation exerts a moderate to low effect on explosive performance [7]. The lack of improvement in explosive power may explain the absence of effects on vertical jump and initial sprint speed. However, this study demonstrates that caffeinated gum consumption can effectively improve 400-meter sprint performance by preserving speed during the final 300–400 meter segment.

## 5. Strengths & limitations

This study offers several novel contributions. Previous studies investigating the effects of caffeine gum have been limited by the absence of caffeine concentration and sympathetic nerve activity measurements. For example, a previous study by Liu et al. (2024) suggested that chewing caffeinated gum reduced the fatigue index [15], but there was a lack of measurement of caffeine concentration in the body and indicators of the mechanisms involved, so the exact physiological mechanisms of caffeine could not be known. This study addresses this gap by quantifying both parameters. Our findings demonstrate that caffeine gum ingestion increases sympathetic nerve activity during exercise, as indicated by elevated α-amylase levels, leading to reduced fatigue and improved 400-meter sprint performance.

Several limitations should be acknowledged in this study. Firstly, the high performance liquid chromatography (HPLC) method was not used to measure caffeine concentration in plasma, which may have resulted in a less accurate calculation of caffeine concentration. However, it has been shown in the literature that caffeine concentration in saliva can reflect the caffeine concentration in plasma [24]. Therefore, we contend that this data remains sufficiently representative. Secondly, the absence of heart rate variability (HRV) data during exercise precludes a comprehensive assessment of autonomic nervous system activity. Nevertheless, the observed increase in α-amylase, a reliable marker of sympathetic nerve activity, provides strong evidence for enhanced sympathetic nerve activity in the CAF trial. Thirdly, the RAST and the 400 m sprint were conducted on the same day in this study. Although a sufficient rest period was provided before the start of the 400 m sprint, future research could explore the potential effects of a different experimental protocol on study results.

## 6. Conclusion

This study demonstrated that pre-exercise caffeine gum supplementation (3 mg/kg) effectively enhance 400-meter sprint performance. The observed improvements are attributed to a reduced fatigue index and increased capacity to maintain speed, particularly in the final 300 to 400 meters. This enhanced performance can be attributed to the augmented sympathetic nervous activity, presumably elicited by caffeine gum administration. Further research could investigate the comparative effects of caffeine gums and capsules on sympathetic nerve activation and subsequent performance outcomes.

## Data Availability

All relevant materials are presented in the present manuscript.
